# Supplementary material for: Characterizing pre-transplant and post-transplant kidney rejection risk by B cell immune repertoire sequencing
Source: Nat Commun. 2019 Apr 23;10:1906. doi: 10.1038/s41467-019-09930-3 (PMC6479061; doi:10.1038/s41467-019-09930-3)
Supplement: Supplementary file 1 — Supplementary Information [file 41467_2019_9930_MOESM1_ESM.pdf]

Supplementary Information

Characterizing pre-transplant and post-transplant kidney rejection risk by B cell immune repertoire sequencing

Pineda et al.

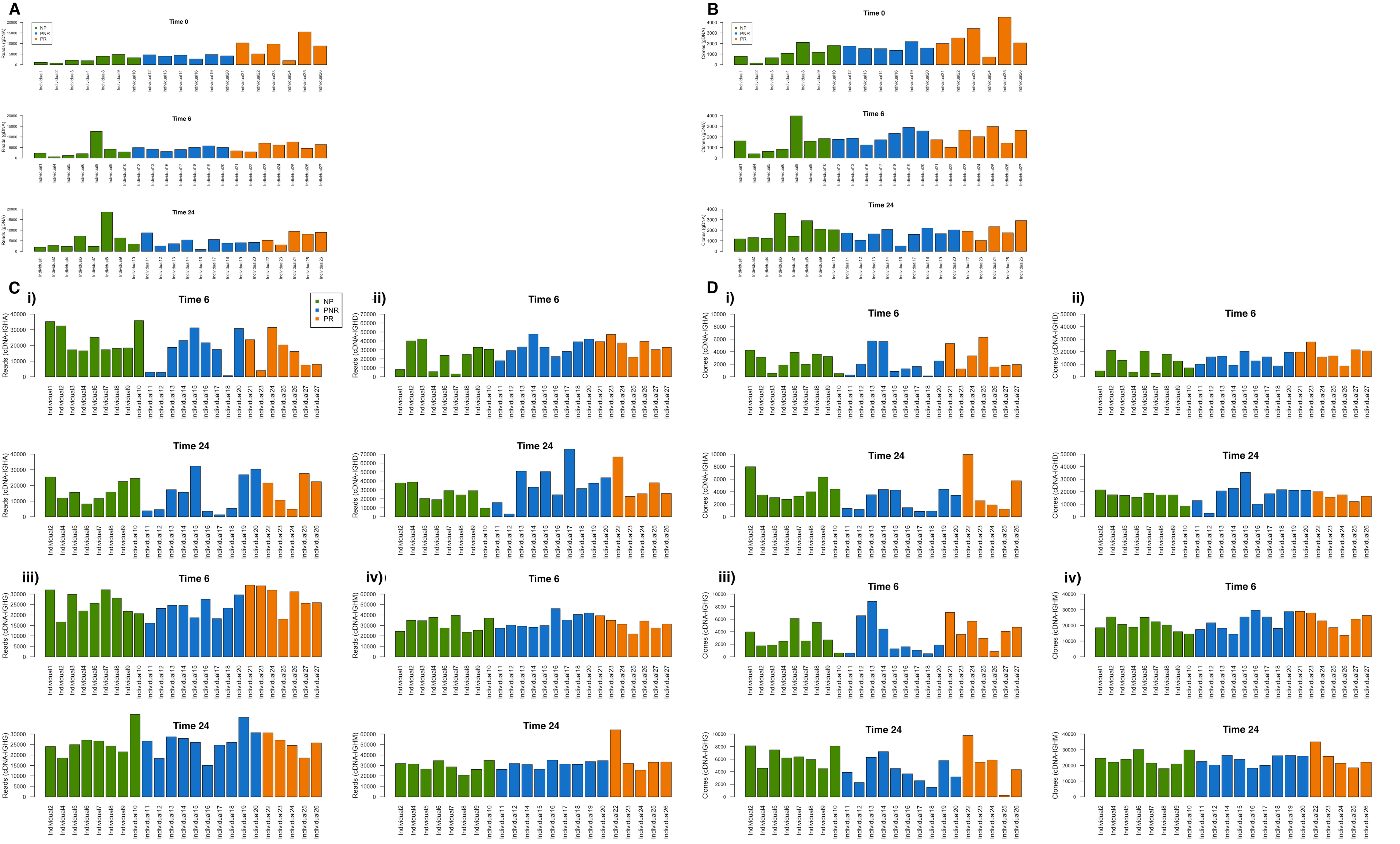

Supplementary Figure 1. Barplots showing the number of reads (A) and number of clones (B) from the gDNA B cell sequencing output and number of reads (C) and number of clones (D) from the cDNA B cell sequencing output. Each bar shows the number of reads or clones per individual after QC at time 0, 6 and 24 for gDNA and time 6 and 24 for cDNA (No samples had available RNA at time 0). The cDNA output is represented by isotypes: IgA (i), IgG (ii) IgD (iii) and IgM (iv). All samples show here passed the 100 clones minimum threshold.

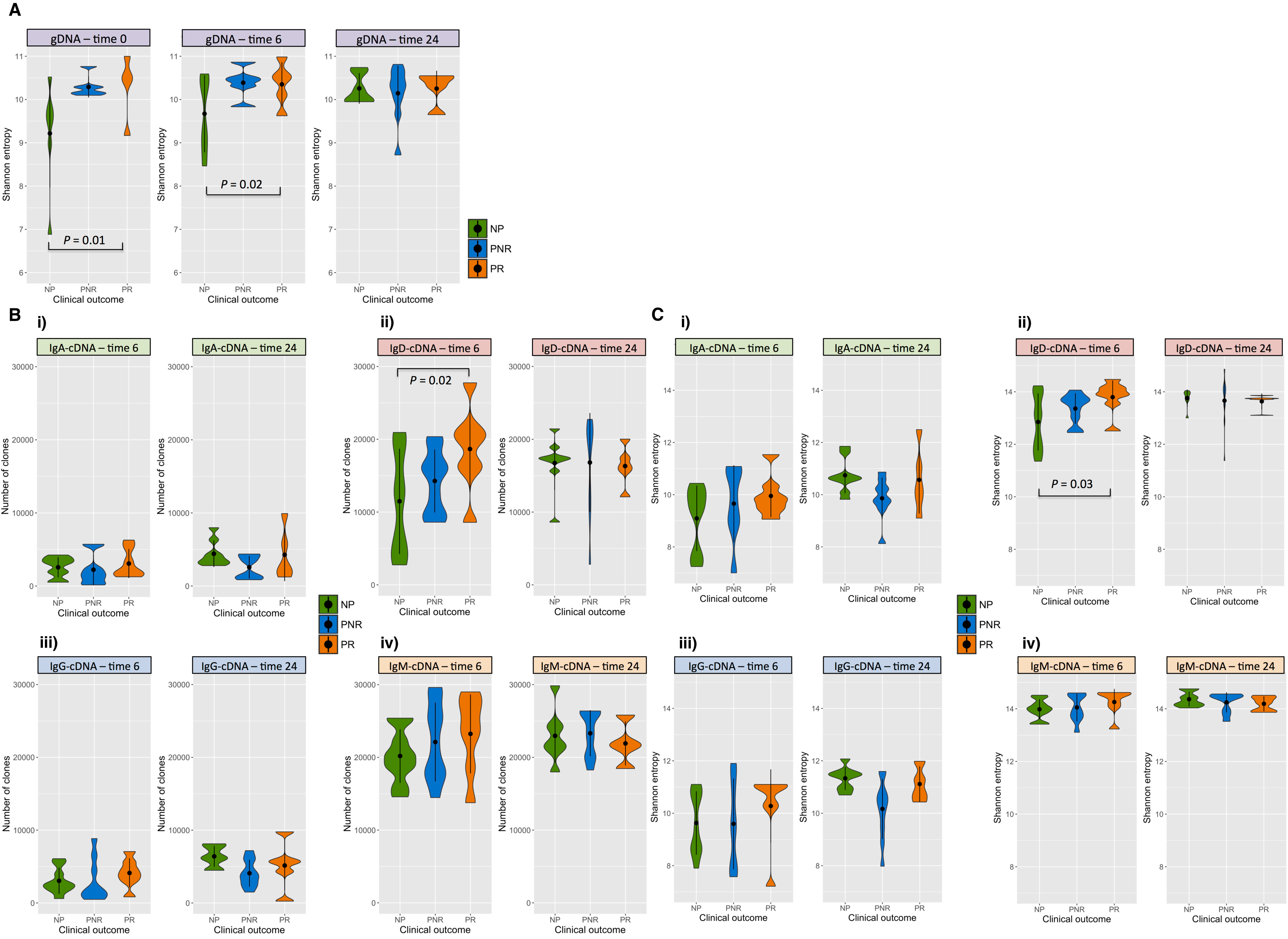

Supplementary Figure 2. Violin plots showing the diversity measured by Shannon Entropy for gDNA (A) at time 0, 6 and 24 months, by number of clones for cDNA (B) and by Shannon Entropy for cDNA (C) at time 6 and 24 months defining the repertoire across the three clinical outcomes (NP, PNR and PR). The cDNA output is represented by isotypes: IgA (i), IgD (ii) IgG (iii) and IgM (iv). The p-values are obtained from the adjustment of a linear model considering the number of clones as a dependent variable and clinical outcome as an independent factor variable. Only p-values<0.05 are shown.

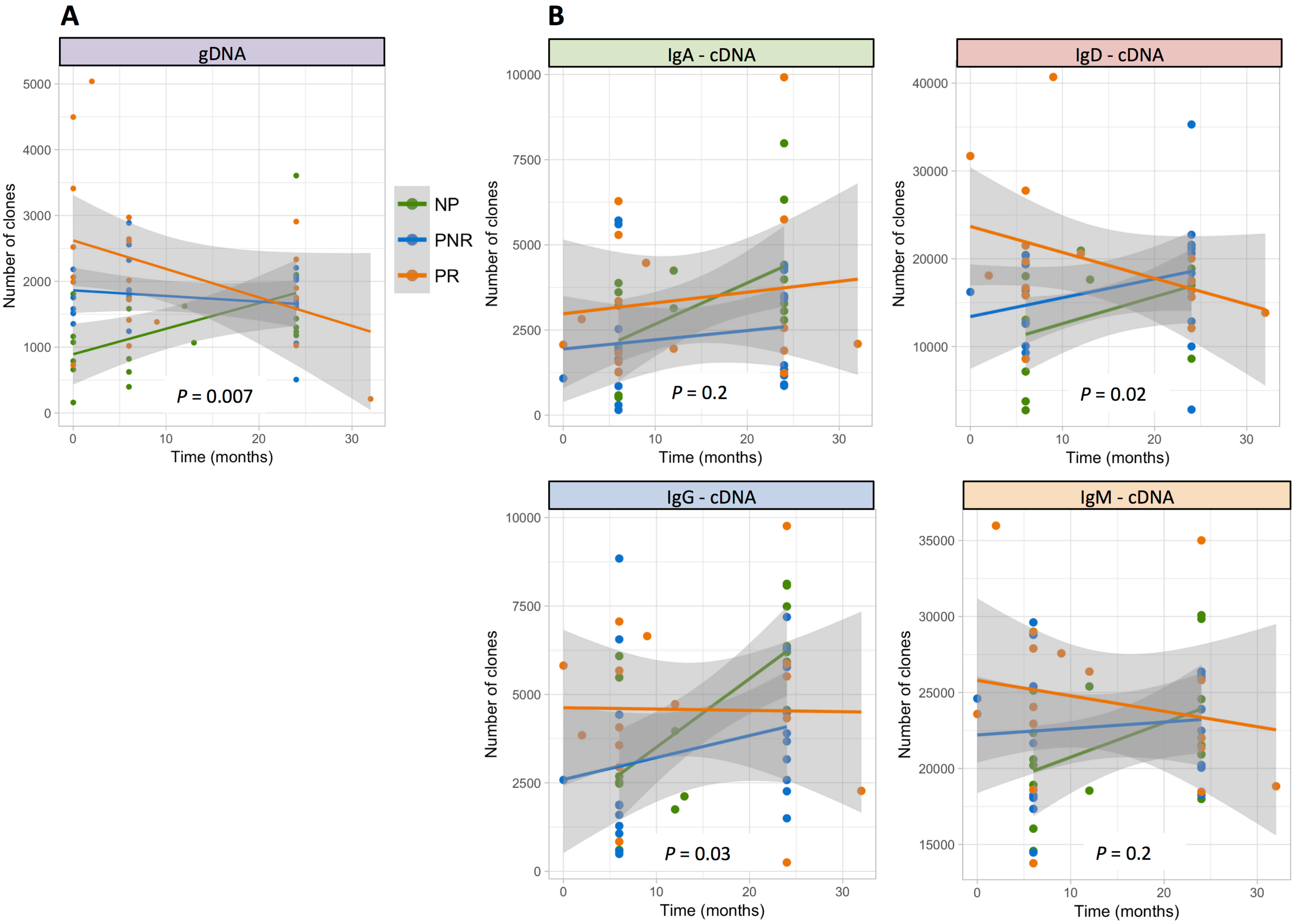

Supplementary Figure 3. Longitudinal data plotted with the fitted line for each clinical outcome. (A) Richness measured by number of clones represented across time points by the three clinical outcomes (NP, PNR, PR) in gDNA. (B) Richness measured by number of clones represented across time points by the three clinical outcomes (NP, PNR, PR) by isotypes in cDNA. The p-values correspond to the interaction term defined by time*clinical outcome adjusting a linear mixed effect model.

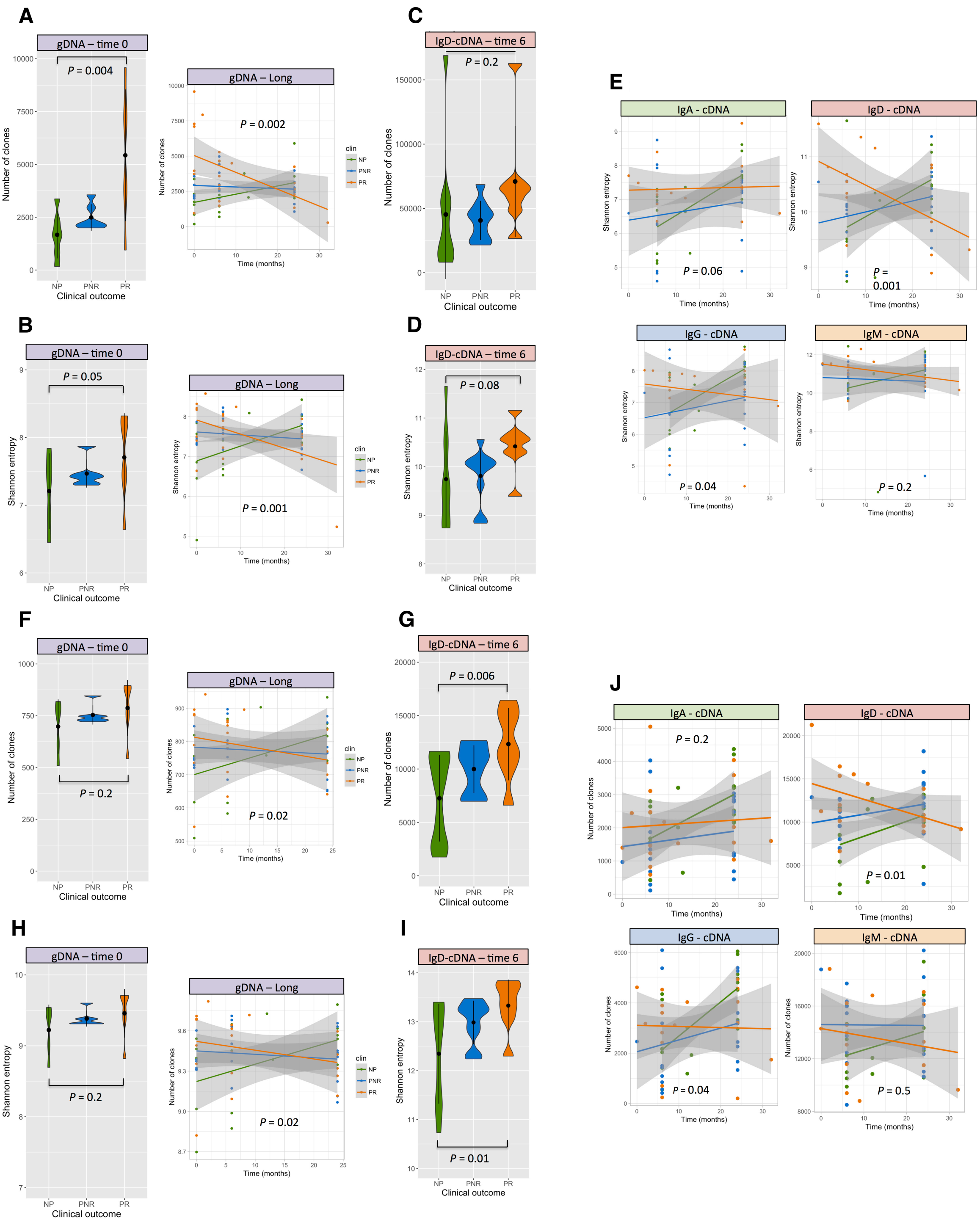

Supplementary Figure 4. Diversity analysis results using Recon (A-E) and using down-sampling (F-J). Violin plots showing the number of clones (richnes) at time 0 and the longitudinal data plotted with the fitted line for each clinical outcome measured by richness (number of clones) for gDNA using recon (A) and using down-sampling (F). Violin plots showing diversity measured by Shannon Entropy at time 0 for gDNA using recon (B) and using down-sampling (H). Violin plots showing the number of clones (richnes) at time 6 for IgD isotype for cDNA using recon (C) and using down-sampling (G). Violin plots showing diversity measured by Shannon Entropy at time 6 for IgD isotype for cDNA using recon (D) and using down-sampling (I). Longitudinal data plotted with the fitted line for each clinical outcome measured by Shannon entropy (diversity) for all isotypes in cDNA using recon (E) and using down-sampling (J). To perform the down-sampling strategy we had to filter out 9 samples with < 1000 clones for gDNA, therefore the sample size for the single time points analysis is reduced losing statistical power.

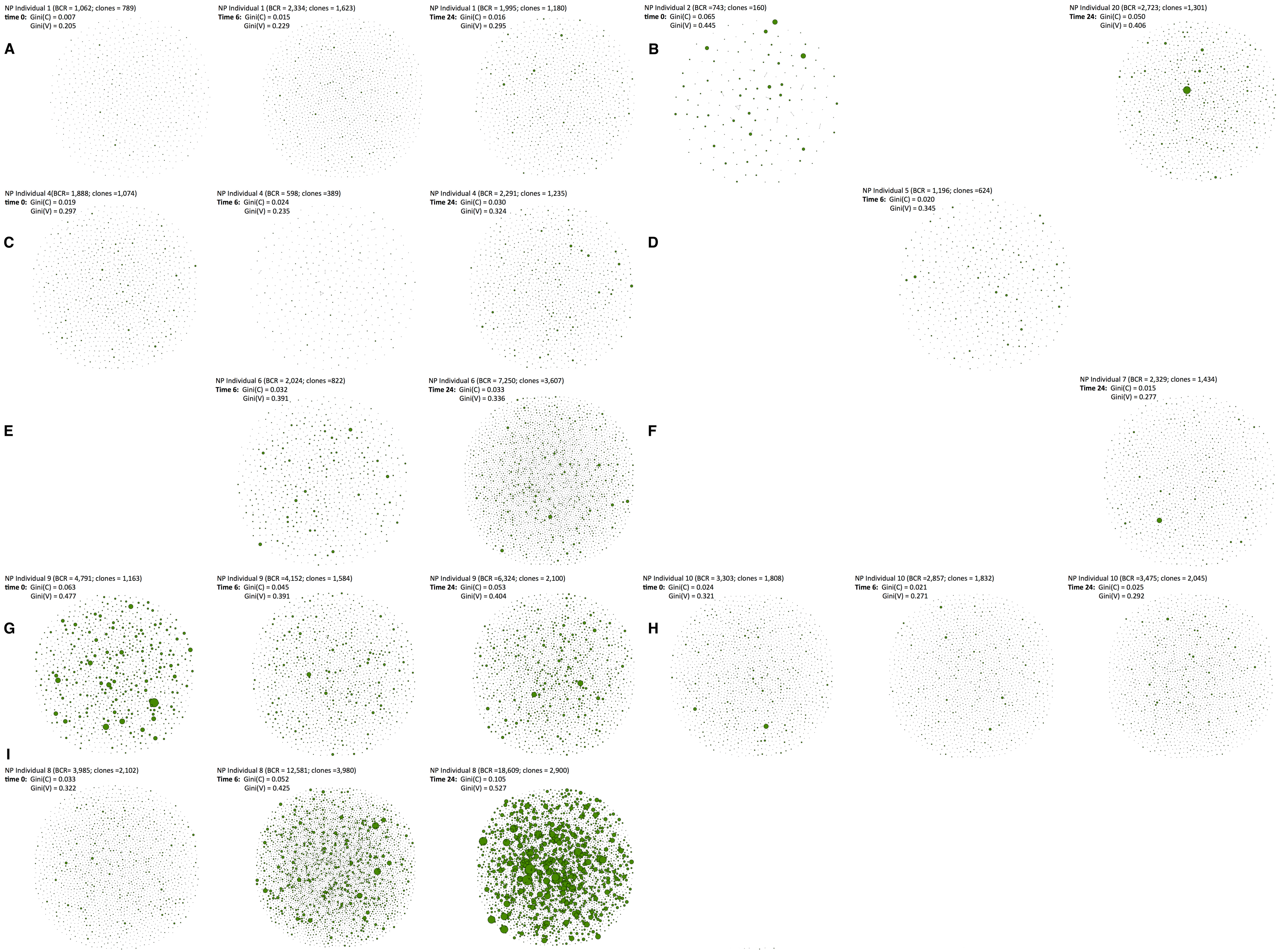

Supplementary Figure 5. Network representation of B cell repertoires from all individuals representing the NP group across time points. Each vertex represents a unique BCR being the vertex size defined by the number of identical BCRs considering the nucleotide sequences. An edge exists between vertices when they belong to the same clone as defined before, so clusters are groups of interconnected vertices forming a clone. Each sample shows the gini index obtained for the vertex size (*Gini(V)*) and cluster size (*Gini(C)*). BCR reflects the total B cell receptors for that specific sample and clones reflect the total number of unique clones. The data that is not represented are those that did not passed the quality control threshold (clones >100). Individual 8 (I) is the patient who developed EBV+ post-transplant lymphoproliferative disease (PTLD) at 2.2 yrs post Ktx, characterized by proliferation of Epstein-Barr virus (EBV)-infected B cells.

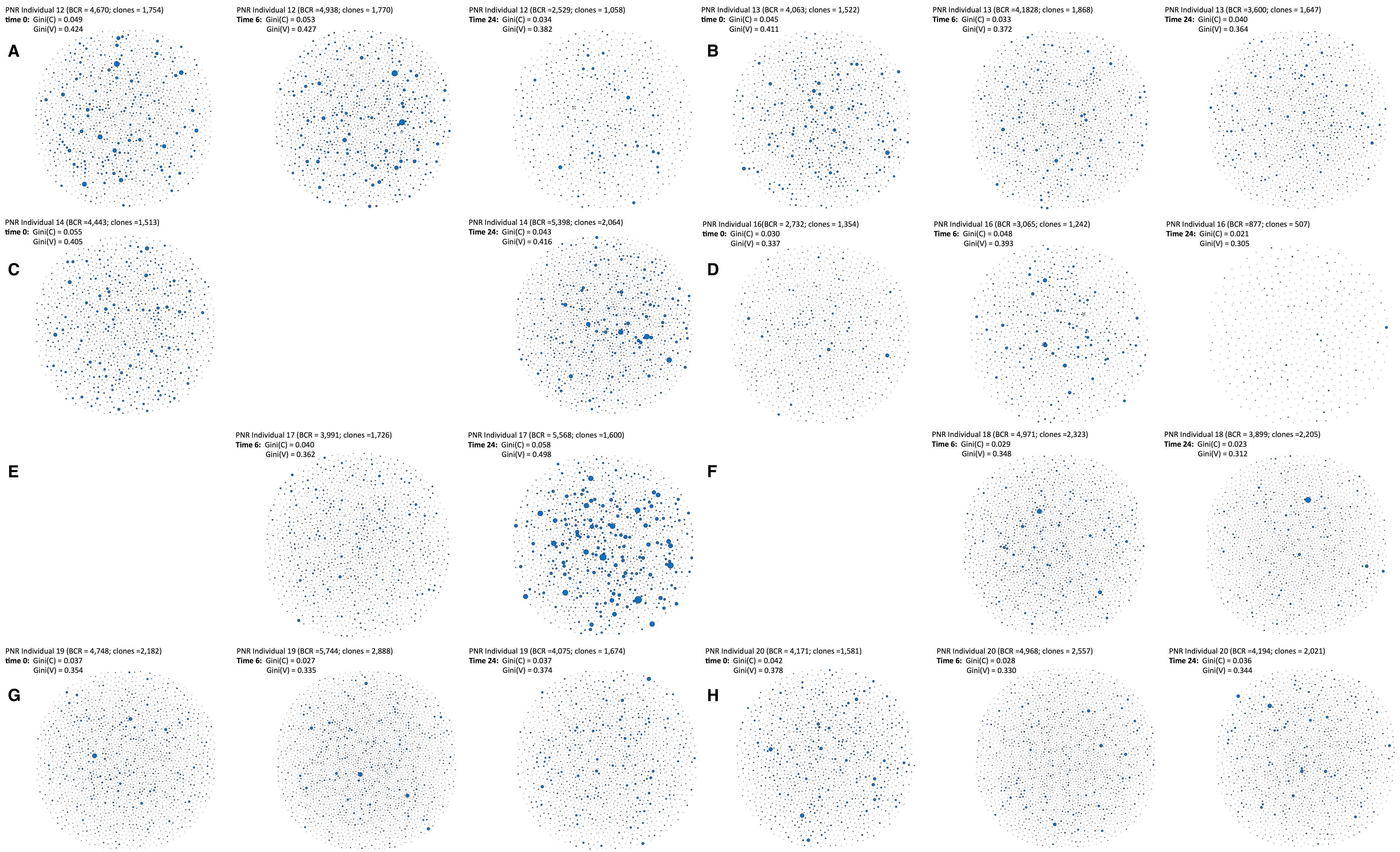

Supplementary Figure 6. Network representation of B cell repertoires from all individuals representing the PNR group across time points. Each vertex represents a unique BCR being the vertex size defined by the number of identical BCRs considering the nucleotide sequences. An edge exists between vertices when they belong to the same clone as defined before, so clusters are groups of interconnected vertices forming a clone. Each sample shows the gini index obtained for the vertex size (*Gini(V)*) and cluster size (*Gini(C)*). BCR reflects the total B cell receptors for that specific sample and clones reflect the total number of unique clones. The data that is not represented are those that did not passed the quality control threshold (clones >100).

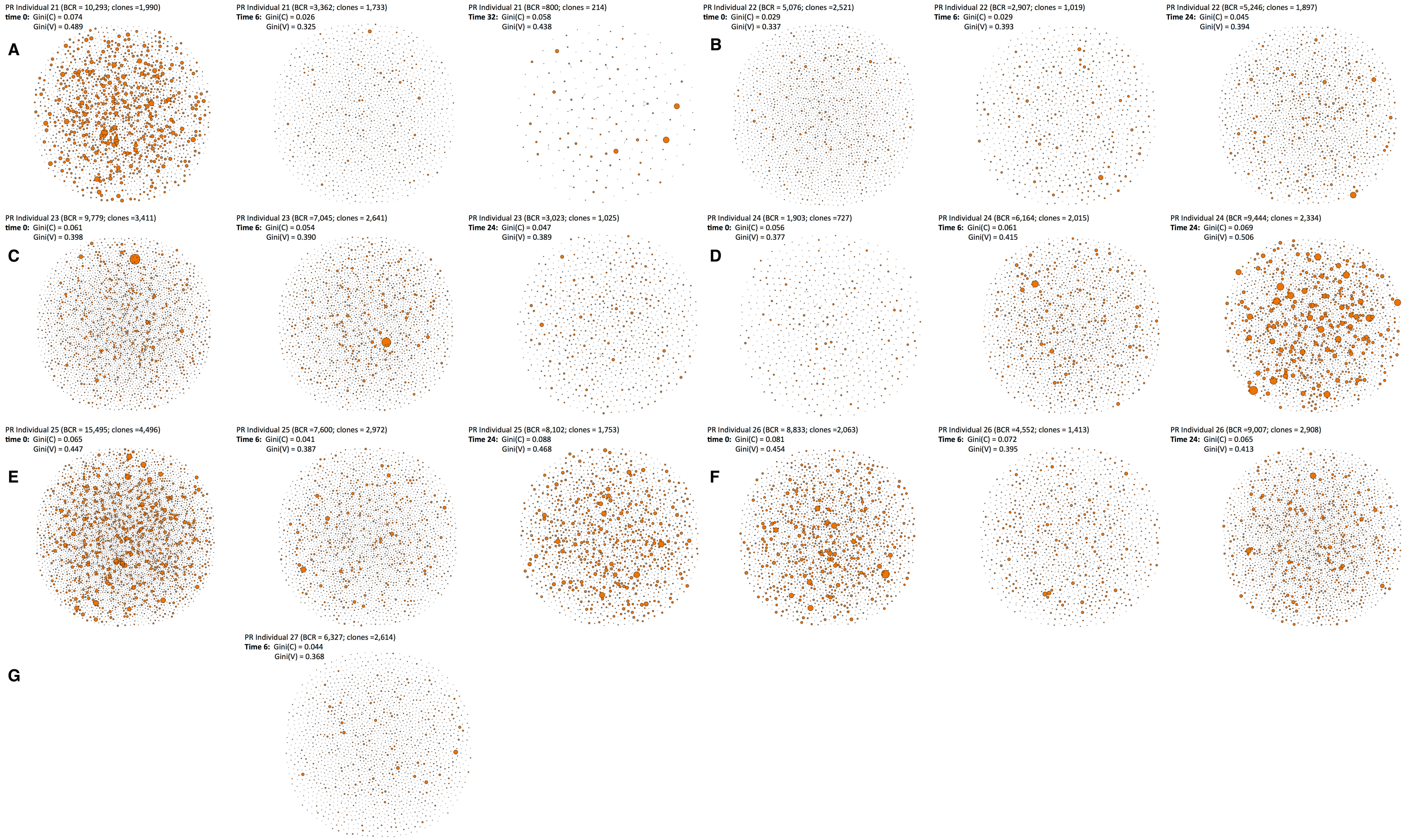

Supplementary Figure 7. Network representation of B cell repertoires from all individuals representing the PR group across time points. Each vertex represents a unique BCR being the vertex size defined by the number of identical BCRs considering the nucleotide sequences. An edge exists between vertices when they belong to the same clone as defined before, so clusters are groups of interconnected vertices forming a clone. Each sample shows the gini index obtained for the vertex size (*Gini(V)*) and cluster size (*Gini(C)*). BCR reflects the total B cell receptors for that specific sample and clones reflect the total number of unique clones. The data that is not represented are those that did not passed the quality control threshold (clones >100).

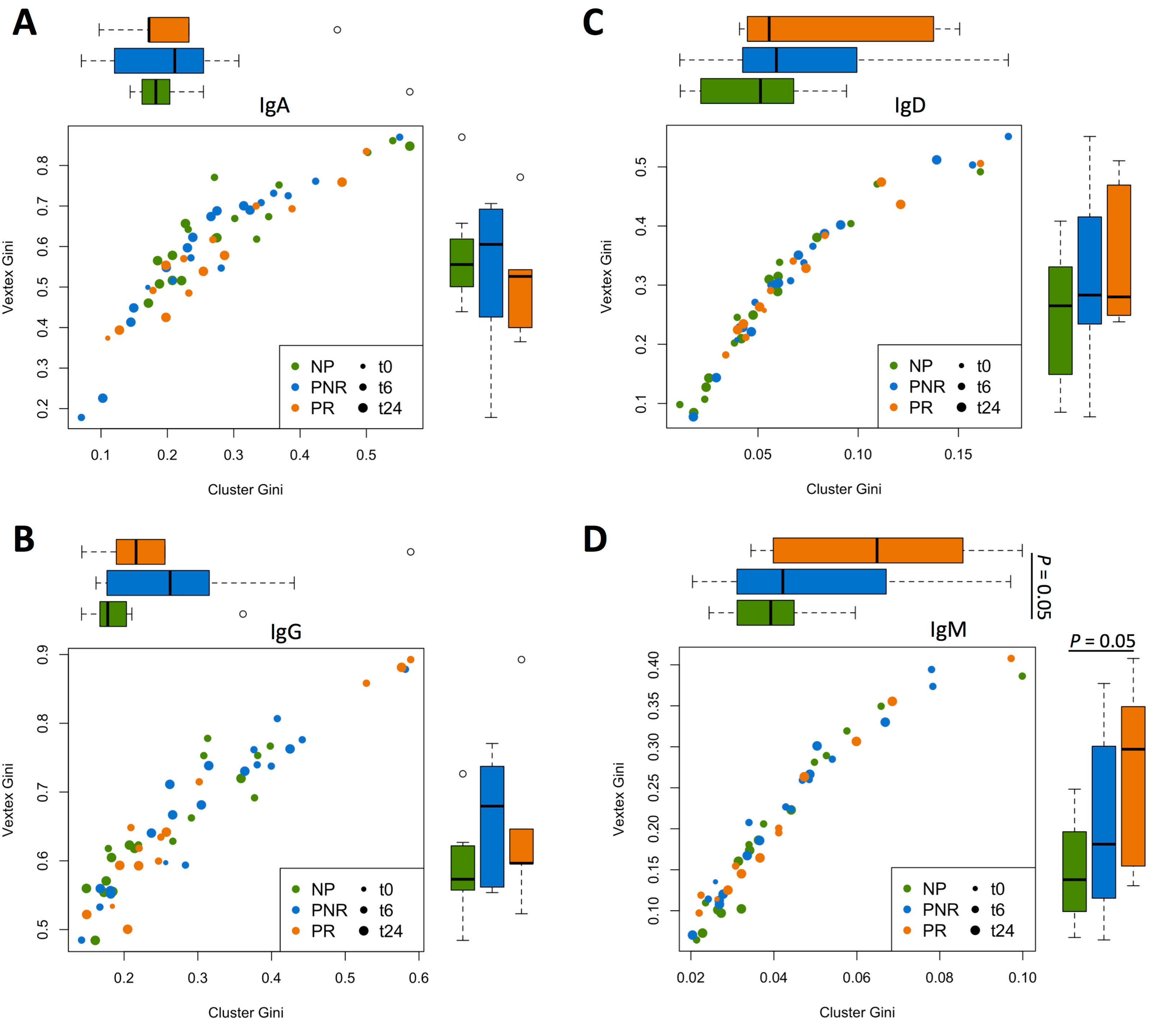

Supplementary Figure 8. Vertex Gini Index plotted against Cluster Gini Index for all the individuals in NP, PNR and PR differentiated by time points at the four isotypes from cDNA (A:IgA, B: IgD, C: IgD and D:IgM). Boxplots shows the *Gini(V)* and *Gini(C)* differences at time 24. The p-values are obtained from the adjustment of a linear model considering the *Gini(V)* and *Gini(C)* as a dependent variable and clinical outcome as an independent factor variable for each time point. Only p-values<0.05 are shown.

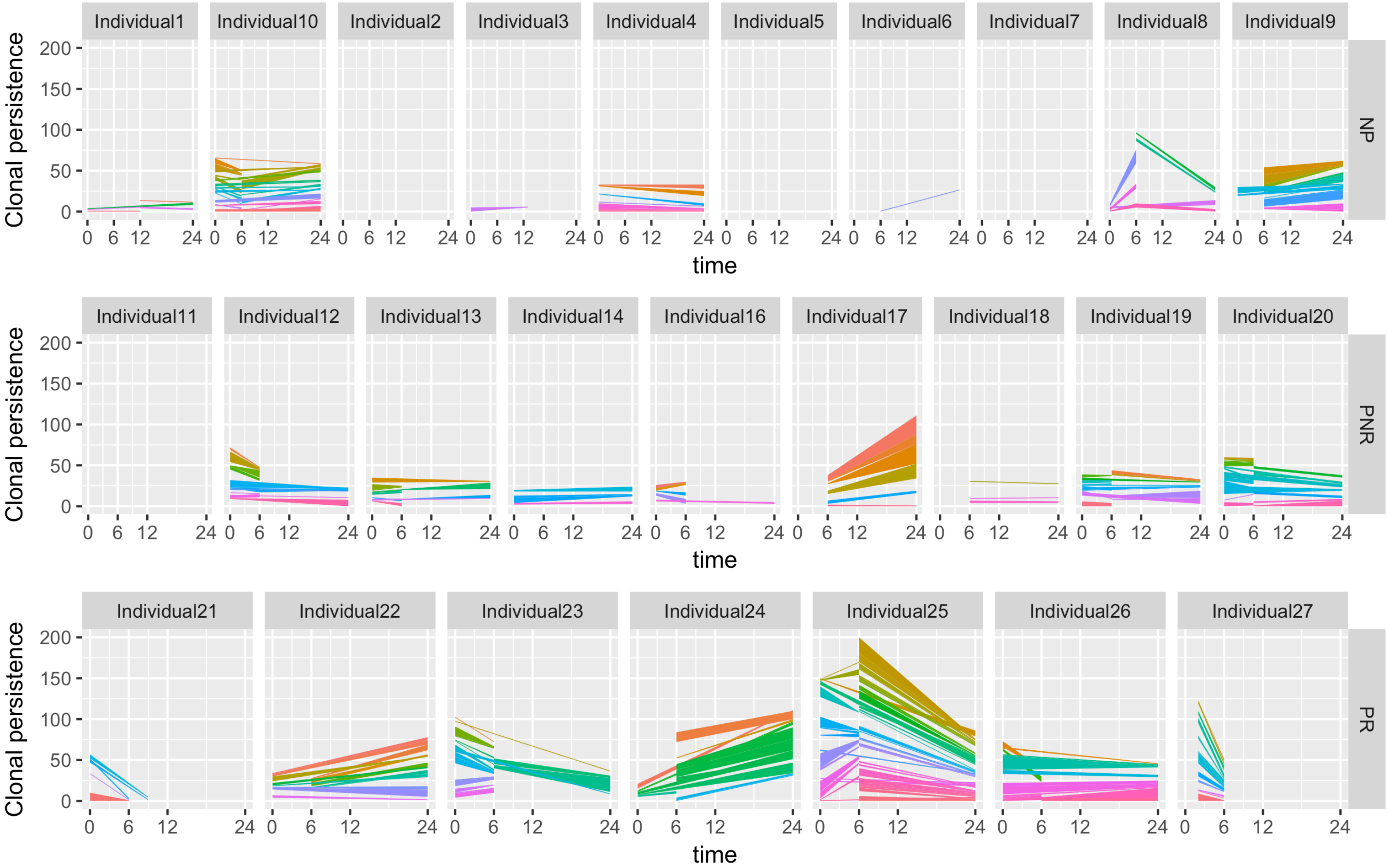

Supplementary Figure 9. Stacked area charts showing the clonal persistence for each individual and clinical outcome. Colors correspond to different clones and the size on the y-axis reflects the counts of each clone. More persistent clones in PR than in NP is observed (p-value = 0.01 calculated using a linear model with the counts of each clone as dependent variable and clinical outcome as the predictor variable). Higher number of persistent clones in PR compared with NP is observed (p-value = 0.09 calculated using linear model with the number of persistent clones as dependent variable and clinical outcome as the predictor variable).

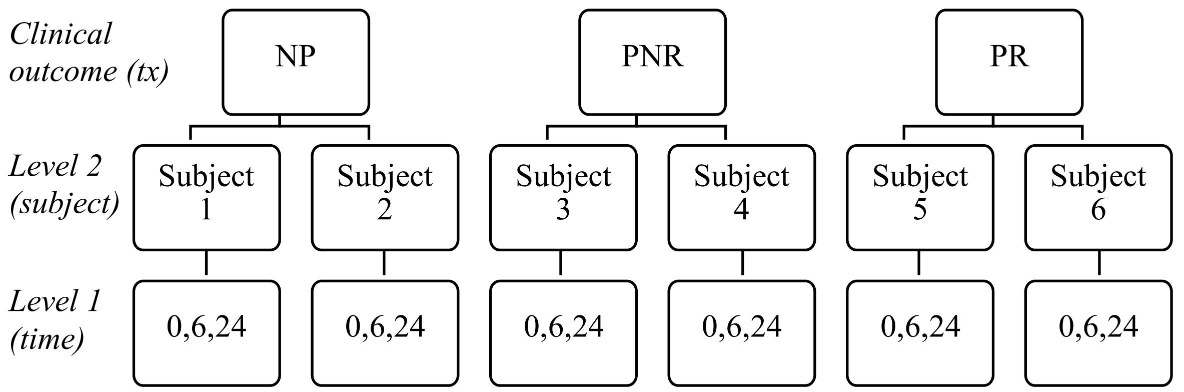

$$Y\ (richnes\ or\ entropy)$$

$Level\ 1$

$$Y = \beta_{0j} + \beta_{ij} time_{ij} + \varepsilon$$

$$\varepsilon \sim N(0, \sigma^2)$$

$Level\ 2$

$$\beta_{0j} = \delta_{00} + \delta_{01} tx_j + U_{0j}$$

$$\beta_{1j} = \delta_{10} + \delta_{11} tx_j + U_{1j}$$

$$\begin{pmatrix} U_{0j} \\ U_{1j} \end{pmatrix} \sim N \begin{pmatrix} 0, \tau_{00}^2 \tau_{01} \\ U_{1j}, \tau_{01}^2 \tau_{10} \end{pmatrix}$$

Supplementary Figure 10. Conditional growth model for linear-mixed effect model

Supplementary Table 1. Clones associated with clinical outcome at each time point. The second column shows the number of individuals that had the clone (present) in each clinical outcome. The p-values are obtained from Fisher's exact test.

CDR3 amino acid sequence	Present<br>Absent			p-value	time	IGHV gene	IGHJ gene
	NP	PNR	PR				
ARDRWFDAFDI<br>ARDRGGDAFDI<br>ARDLGFDAFDI<br>ARADTDDAFDI<br>ARVGGWEAFDI<br>ARQLAGYAFDI	0<br>9	0<br>10	3<br>3	0.009	0	*IGHV1-18*	*IGHJ3*
ARTSGWYFDY<br>AREWDDYFDY<br>ARERGYYFDY<br>AREIGYYFDY<br>AREDGDYFDY<br>AREVFNYFDY<br>ARDRGGYFDY<br>ARDEGYYFDY<br>ARDWRYYFDY<br>ARVYGDYEGY<br>ARVRGSYYDY<br>ARYSGSYFDY	0<br>9	0<br>10	3<br>3	0.009	0	*IGHV3-21*	*IGHJ4*
ANSYGDNYYYGMDV<br>AREGIGNYYYGMDV<br>ARESLHYYYYGMDV<br>ARDMGDYYYYGMDV<br>ARDRLYYYYYGMDV<br>ARADDFYYYYGMDV<br>ARGDDYYYYYGMDV<br>ARGGRLGYYYDMDV<br>ARVGNYYYYYGMDV<br>ARVEFYYYYYGMDV<br>ARVGNYYYYCGMDV<br>ASVGNYYYYYGMDV<br>ARKGSYYYYYGMDV<br>ARRGIGYYYYGMDV<br>AGGDDYYYYYGMDV	0<br>9	1<br>9	3<br>3	0.04	0	*IGHV3-48*	*IGHJ6*
AREQYYYYGMDV<br>ARDRDYYYGMDV<br>ARDDLYYYGMDI<br>ARDDLYYYGMDV<br>ARDGSYYYGMDV<br>ARAPGYYYGMDV<br>ARGLDYYYGMDV<br>ARVDYYYYGMDV	0<br>9	1<br>9	3<br>3	0.04	0	*IGHV3-53*	*IGHJ6*
TRDAY<br>AREYY<br>ARDDY	0<br>9	0<br>10	3<br>3	0.009	0	*IGHV3-7*	*IGHJ4*

ARDVH<br>ARGAY<br>ARGGY<br>ARGVY<br>ARLDY					
AKDRYYDSSGYLDY<br>AKARYYDSSGYLDY<br>AKGNYYDSSGYYDY<br>AKGRDYDSSGYFDY<br>AKGYYYDSSGYYDY	1 0 3<br>8 10 3	0.02	0	*IGHV3-9*	*IGHJ4*
AKDRYYYYYYGMDV<br>AKDSGSSYYYGMDV<br>AKDIAGHYYYGMDV<br>AKDMKVVYYYGMDV<br>AKDIEGYYYYGMDV<br>AKDIGSYYGNGMDV<br>AKDPDSNYYYGMDV<br>AKDAMAYYYYGMDV<br>AKDFWAYYYYGMDV<br>AKVGAAYYYYGMDV	0 1 3<br>9 9 3	0.04	0	*IGHV3-9*	*IGHJ6*
ATGTDYYYYYGMDV<br>ARSAASYYYYYMDV<br>ARQPSYYYYYGMDV<br>ARPANYYYYYYMDV<br>ARLANYYYYYYMDV<br>ARDLPNYYYYGMDV<br>ARADYDYYYYGMDV<br>ARGQGYYYYYGMDV<br>ARGQDLYYYYGMDV<br>ARGRGVHYYYGMDV<br>ARGLPNYYYYYMDV<br>ARGGHYYYYYGMDV<br>ARGTGDYYYYGMDV<br>ARVRIHYYYYGMDV<br>ARASRRYYYYGMDV	0 1 3<br>9 10 3	0.04	0	*IGHV4-34*	*IGHJ6*
AREEYYDSSGYSDY<br>ARDNYYDSSGPNDY<br>ARDYYYDSSGSFDY<br>ARAYYYDSSGYYVY<br>ARGDYYDSSGSFDY	1 0 3<br>8 10 4	0.04	6	*IGHV1-2*	*IGHJ-4*
TTDQYYYDSSGYYRRDY<br>TTDPYYYDSSGYYFFDY<br>TTDAYYYDSSGYFYFDY<br>TTDPYYYDSSGYSILDY<br>TTDVHYYDSSGYKYFDY<br>TTDLDYYDSSGYYPFDY<br>TTVAYYYDSSGYYYGHY	0 0 3<br>9 10 4	0.01	6	*IGHV3-15*	*IGHJ4*
ARTSGWYFDY<br>AREWDDYFDY<br>ARERGYYFDY<br>AREIGYYFDY<br>AREDGDYFDY<br>AREVFNYFDY	1 0 3<br>8 10 4	0.04	6	*IGHV3-21*	*IGHJ4*

ARDRGGYFDY<br>ARDEGYYFDY<br>ARDWRYYFDY<br>ARVYGDYEGY<br>ARVRGSYYDY<br>ARYSGSYFDY					
AKDADYYDSSGYFDY<br>AKDAYYYDSSGYLDY<br>AKAPGYYDSSGYFDY<br>AKAPSYYDSSGYVDY<br>ARERNYYDSSGYYDY<br>AREEYYYDSSGYYDY<br>ARDTYYYDSSGAFDY<br>ARDQYYYDSSGYFDY<br>ARDPFYYDSSGYVAY<br>ARDRHYYDNTGYVDY<br>ARDLGYYDSSGSLDY<br>ARDLGYYDSSGYLAY<br>ARDGGYYDSSGHLDY<br>ARDSLYYDSSGYPDY<br>ARDLRYYDSSGYLDY<br>ARAVDYYDSSGYLDY<br>ARGLDYYDSSGTLDY<br>ARVTGYYDSSGYFDY	1 0 3<br>8 10 4	0.04	6	*IGHV3-30*	*IGHJ4_45*
ARDSYYDSSGYYPHYYGMDV<br>AREYYYDSSGYYTYYYGMDV	0 0 2<br>8 10 3	0.04	24	*IGHV1-46*	*IGHJ6*
ASESYSSSFGY	0 0 2<br>8 10 3	0.04	24	*IGHV1-8_*	*IGHJ4*
ARGWYCSSTSCDYDY	0 0 2<br>8 10 3	0.04	24	*IGHV1-8*	*IGHJ4*
ARGLSSGWYDPYPYYYYYYGMDV	0 0 2<br>8 10 3	0.04	24	*IGHV1-8*	*IGHJ6*
ARTNLGYCSGGSCYYWFDP	0 0 2<br>8 10 3	0.04	24	*IGHV2-26*	*IGHJ5*
ARDASLKWGGMD	0 0 2<br>8 10 3	0.04	24	*IGHV3-11*	*IGHJ6*
TTEIFDY<br>TTEIFGY<br>TTEWLDY<br>TTDRTDY<br>TTDAPDY<br>TTAYFDY<br>TTGPLDY	0 0 2<br>8 10 3	0.04	24	*IGHV3-15*	*IGHJ4*
AKERYYYDSSGYYGY<br>AKEADYYDSSGLFDY<br>AKDPGYYDSSGYWDY	0 1 3<br>8 9 2	0.03	24	*IGHV3-23*	*IGHJ4*

AKDPGYYDSSGYYHY<br>AKDPGYYDSSGYCHY<br>AKDPFYYDSSGYWDY<br>AKDRQYYDSSGYFDY<br>AKDLYYYDSSGYYSY<br>AKGQDYYDSSGYYDY<br>AKLTYYYDSSGYSDY					
AKEYYDSSGYYYPFDY<br>AKDYYDSSGYYSLFDY<br>ATYYYDSSGYYYYFDY	0 0 2<br>8 10 3	0.04	24	*IGHV3-23*	*IGHJ4*
AKEGIYGDYNYYYGMDV<br>AKGRVYGDYVYYYGMDV<br>ARASDYGDYYYYYGMDV	0 0 2<br>8 10 3	0.04	24	*IGHV3-23*	*IGHJ6*
AKDTAHHGSGSSWMAPIDY	0 0 2<br>8 10 3	0.04	24	*IGHV3-30*	*IGHJ6*
TKDSSSSNYYFGLDV<br>AKDRGSSFYYYGMDV<br>AKYSSSSNYYYGMDV	0 0 2<br>8 10 3	0.04	24	*IGHV3-30*	*IGHJ6*
ARTGIDY<br>ARDSFDY<br>ARDGHDY<br>ARDGGDY<br>ASDYFDY	0 0 2<br>8 10 3	0.04	24	*IGHV3-33*	*IGHJ4*
TRDRRGGLDY<br>ARTNGSYYDY<br>ARERVGAIDY<br>AREGEGYFDY<br>AREGSGLFDY<br>AREGGSYFDY<br>AREVGGSIDY<br>ARDRLDYFDY<br>ARDRGGYFDY<br>ARDSGGGFDY<br>ARDIMGYFDY<br>ARDHSGSFDY<br>ARDRGIHFDY<br>ARDRGDSFDY<br>ARDRNGEFDY<br>ARDRGYYFDY<br>ARDREGYFDY<br>ARDVWYYFDY<br>ARDYRYYFDY<br>ARDWAYYFDY<br>ARGSGSYLDY<br>ASDYGVNFDY<br>ASYYGVNFDY	0 2 3<br>8 8 2	0.04	24	*IGHV3-33*	*IGHJ4*
TRVSGPYGSGSQDY	0 0 2<br>8 10 3	0.04	24	*IGHV3-49*	*IGHJ4*
ARDRYCSGGSCSHFDY<br>ARVGYCSGGSCNDLDY	0 0 2	0.04	24	*IGHV3-7*	*IGHJ4*

ARVGYCSGGSCNDFDY<br>ASEGYCSGGSCYWFDY	8 10 3				
ARVDDSSGYYLYYFDY<br>ARVDDSSGYYLCYFDY<br>ARVDDSSGYYQYYFDY	0 0 2<br>8 10 3	0.04	24	*IGHV3-7*	*IGHJ4*
ARDRSYGMDV<br>ARDLSYGMHV<br>ARGNYYGMDV<br>ARGLAYGMDV<br>ARGIYYAMDV<br>ARGHYYGMDV<br>ARVGYYGMDV	0 0 2<br>8 10 3	0.04	24	*IGHV3-74*	*IGHJ6*
ARERTVTTPAYYYGTDV<br>ARERTVTTPAYYYGMDV<br>ARDSTVTTGYYYYGMDV	0 0 2<br>8 10 3	0.04	24	*IGHV4-31*	*IGHJ6*
ARVNVVVPSGSYSG	0 0 2<br>8 10 3	0.04	24	*IGHV4-39*	*IGHJ5*
ARDRRDYGDYKYYFDY	0 0 2<br>8 10 3	0.04	24	*IGHV4-4*	*IGHJ4*

Supplementary Table 2. List of persistence clones shared across individuals

CDR3 amino acid sequence	Clinical Outcome (shared individuals)		
	NP	PNR	PR
ARDRRWSFDY<br>ARDRRWPFDY<br>ARDLSWAFDY<br>VRDYLWGFDY<br>VRDYYYRFDY<br>ARDSVYAFDY<br>VRDRDWGFDF<br>ARDLLGAFDY<br>ARDHNWAFDY<br>ARDVNWAFDY<br>ARAYGGNYDY<br>ARDWNWAFDY<br>VRDYYYRFDY<br>ARDHDWAFDC<br>VRDFDWNFDY<br>ARDRGHYFDY	Individual 9	Individual 12, 13, 14,16, 19	
ARVQGHYYYYGMDV<br>ARTLNAYYYYGMDV<br>ARDRRDYYYYGMDV<br>AKEIRGYYYYGMDV<br>AGNYGDYDYYGMDV<br>ARDLSYYYYYGMDV<br>AKDPAWDYYYGMDV<br>AKDIGVYYYYGMDV<br>AKAMADYYYYGMDV<br>AKDKGSYYYYGMDV<br>ARETGDYYYYGMDV<br>AKDLTDYYYYGMDV<br>ARARGDYYYYGMDV<br>ARDSHYEYYYGMDV<br>AKVRATLYYYGMDV	Individual 10	Individual 12, 14, 19, 20	Individual 27
ARDYYDYGMDV<br>ARDSGDYGMDV<br>AREDGEYGMDV<br>ARVSGSYGMDV	Individual 9	Individual 13	
ARGSRKYYYYYGMDV<br>ARGRLRVYYYYGMDV<br>ARGEGDYNYYYGMDV<br>ARGVILYYYYYGMDV<br>ARGNGRYYYYYGMDV	Individual 9	Individual 18	
ARDVWYYFDY<br>ARERVGAIDY<br>ARTNGSYYDY		Individual 12, 19	Individual 24

ARDSGGGFDY<br>ARDRLDYFDY<br>ARDRGGYFDY<br>ARDRGDSFDY			
ARAINYYYGMDV<br>ARDYYYYYGMDV<br>ASNYYYYYGMDV<br>AREEGYYYGMDV<br>ARDQDYYYGMDV<br>ARDRSYYYGMDV		Individual 19, 20	Individual 26
AKAPSYYDSSGYVDY<br>ARGLDYYDSSGTLDY<br>ARDSLYYDSSGYPDY<br>ARDLRYYDSSGYLDY<br>ARDLGYYDSSGSLDY<br>AREEYYYDSSGYYDY<br>AKDAYYYDSSGYLDY<br>ARERNYYDSSGYYDY<br>ARAVDYYDSSGYLDY<br>ARDLGYYDSSGYLAY	Individual 9	Individual 20	Individual 25
ARDQRFYFDY<br>AKDSGSYYDY<br>AKDNGDYFDY<br>AKDRDTCFDY<br>AKDYGFYFDY<br>AKDRGFGFDY<br>AKGYSGSLDY<br>AKDRVHYFDY<br>AKDRGPYFDY<br>AKYRGPYFDY		Individual 20	Individual 22, 26
ARGRRDYYYGMDV<br>ARGYYYYYYGMDV<br>ARGRYYYYYGMDV<br>ARGYHYYYYGMDV<br>ARVYYYYYYGMDV<br>ARRSDYYYYGMDV<br>ARGAFYYYYGMDV		Individual 14	Individual 23, 25
AKLSGYYYYYGMDV<br>AKASGYYYYYGMDV<br>AKLLDYYYYYGMDV<br>AKVRGPYYYYGMDV<br>ARDPEGYYYYGMDV	Individual 10		Individual 23
ARLYDYGDYYYYYGMDV<br>ARDRSGYEYYYYYGMDV<br>ARASDYGDYYYYYGMDV<br>ARDRRDGDYYYYYGMDV<br>AREGGGYDFYYYYGMDV<br>ARDRGYDGYYYYYGMDV<br>ARYGYGDYGYYYYGMDV<br>ARDRGDYVLGYYYGMDV	Individual 9		Individual 23, 26, 27

AKDIAYYFDY<br>AKSRDYYFDY<br>AKDEIYYFDY<br>AKDLYGYFDY<br>AKDRWNYVDY			Individual 22, 24
ARYNYDSSGYCDY<br>AREDYDSSGYTDY<br>AKNYYDSSGYLRY<br>ARDYYDSSGYFDY	Individual 10		Individual 25
ARDTPNYYYYGMDV<br>ARDRPRYYYYGMDV<br>ARAAPYYYYYGMDV<br>AREIHYYYYYGMDV		Individual 14	Individual 25
ARVFEYYFDY<br>ARGSGWLVDY<br>AGGSGWIFTN<br>ARGFGDYFDY<br>AAGSGWLIDN<br>ARDSSGSSDY			Individual 22, 25
ARHRQYYYYYGMDV<br>ARRLGGYYYYGMDV<br>ARGDRYYYYYGMDV<br>ARQPSYYYYYGMDV		Individual 12	Individual 25
AKEADYYDSSGLFDY<br>AKDRQYYDSSGYFDY<br>AKGQDYYDSSGYYDY<br>AKDPGYYDSSGYYHY<br>AKDPGYYDSSGYWDY<br>AKDPGYYDSSGYCHY		Individual 23, 25	
ARQAGSSFDY<br>ARRVRGNFDY<br>ARGGTYYFDY<br>ARDWGGHFDY		Individual 20	Individual 26
ARQPSYYYYYGMDV<br>ARGQGYYYYYGMDV<br>ARGGHYYYYYGMDV<br>ARSAASYYYYYMDV<br>ARGRGVHYYYGMDV<br>ARPANYYYYYYMDV<br>ARLANYYYYYYMDV<br>ARGTGDYYYYGMDV			Individual 25, 26
ARFYYYYYGMDV<br>ARISTYYYGMDV<br>ASGRNYYYGMDV<br>ARWSYYYYYMDV		Individual 20	Individual 26
AREARLYYYYGMDV<br>ARDLDYYYYYGMDV<br>ARSAGHYYYYGMDV<br>ARVLRYYYYYGMDV	Individual 10		Individual 27

ARDHYYDSSGYLDY<br>ARCYYYDSSGPIDY<br>AKDSYYDSSGPFDY<br>AKAYYYDSSGYFNY<br>ASSDYYDSSGYLDY<br>AKDYYYDSSGFLGY<br>ARDLYYDSSGYFDY		Individual 20	Individual 27
ARDGYKTYYYYGMDV<br>ARDVGAAYYYYGMDV<br>ARAHGDLYYYYGMDV<br>ARDAGSYYYYYGMDV<br>ARAGGLYYYYYGMDV<br>ARSGGPYYYYYGMDV<br>AKDRELDYYYYGMDV		Individual 20	Individual 27
