# Supplementary material for: Characterizing pre-transplant and post-transplant kidney rejection risk by B cell immune repertoire sequencing
Source: Nat Commun. 2019 Apr 23;10:1906. doi: 10.1038/s41467-019-09930-3 (PMC6479061; doi:10.1038/s41467-019-09930-3)
Supplement: Supplementary file 3 — Reporting Summary [file 41467_2019_9930_MOESM3_ESM.pdf]

nature research

Corresponding author(s): silvia.pinedasanjuan@ucsf.edu

Last updated by author(s): 1/28/2019

Reporting Summary

Nature Research wishes to improve the reproducibility of the work that we publish. This form provides structure for consistency and transparency in reporting. For further information on Nature Research policies, see Authors & Referees and the Editorial Policy Checklist.

Statistics

For all statistical analyses, confirm that the following items are present in the figure legend, table legend, main text, or Methods section.

n/a	Confirmed	
☐	☒	The exact sample size (n) for each experimental group/condition, given as a discrete number and unit of measurement
☐	☒	A statement on whether measurements were taken from distinct samples or whether the same sample was measured repeatedly
☐	☒	The statistical test(s) used AND whether they are one- or two-sided<br>*Only common tests should be described solely by name; describe more complex techniques in the Methods section.*
☐	☒	A description of all covariates tested
☐	☒	A description of any assumptions or corrections, such as tests of normality and adjustment for multiple comparisons
☐	☒	A full description of the statistical parameters including central tendency (e.g. means) or other basic estimates (e.g. regression coefficient) AND variation (e.g. standard deviation) or associated estimates of uncertainty (e.g. confidence intervals)
☐	☒	For null hypothesis testing, the test statistic (e.g. F, t, r) with confidence intervals, effect sizes, degrees of freedom and P value noted<br>*Give P values as exact values whenever suitable.*
☒	☐	For Bayesian analysis, information on the choice of priors and Markov chain Monte Carlo settings
☐	☒	For hierarchical and complex designs, identification of the appropriate level for tests and full reporting of outcomes
☐	☒	Estimates of effect sizes (e.g. Cohen's d, Pearson's r), indicating how they were calculated

Our web collection on statistics for biologists contains articles on many of the points above.

Software and code

Policy information about availability of computer code

Data collection	Genomic DNA from whole blood clot was extracted by using Clotspin® Baskets and the Gentra® Puregene®Blood Kit (Qiagen, Valencia, CA). RNA quantity and integrity were determined with the Thermo Scientific NanoDrop ND-2000 UV-Vis Spectrophotometer and Agilent Bioanalyzer. For gDNA sequence AmpliTaq Gold (Roche) was used and for cDNA MiSeq. Paired-end reads were merged using FLASH and the alignment program used was IgBLAST. Everything is detailed in material and methods.
Data analysis	For data analysis we used R for the statistical analysis. We used by defect libraries for ANOVA test and Chisq test and lmer for the longitudinal analysis, igraph for the network plots, ineq for the gini index and pheatmap for the IGHV gene representation. We used Recon (reconstruction of estimated clones from observed numbers) for the missing-species problem. Everything is detailed in material and methods.

For manuscripts utilizing custom algorithms or software that are central to the research but not yet described in published literature, software must be made available to editors/reviewers. We strongly encourage code deposition in a community repository (e.g. GitHub). See the Nature Research guidelines for submitting code & software for further information.

Data

Policy information about availability of data

All manuscripts must include a data availability statement. This statement should provide the following information, where applicable:

- Accession codes, unique identifiers, or web links for publicly available datasets
- A list of figures that have associated raw data
- A description of any restrictions on data availability

The data supporting this publication has been deposited in the ImmPort repository under the study accession DSY1361 as specified in the manuscript.

Field-specific reporting

Please select the one below that is the best fit for your research. If you are not sure, read the appropriate sections before making your selection.

☒ Life sciences ☐ Behavioural & social sciences ☐ Ecological, evolutionary & environmental sciences

For a reference copy of the document with all sections, see nature.com/documents/nr-reporting-summary-flat.pdf

Life sciences study design

All studies must disclose on these points even when the disclosure is negative.

Sample size	83 samples for gDNA and 55 samples for cDNA from 27 patients
Data exclusions	we discounted samples with less than 100 clones (69 samples from gDNA and 55 samples from cDNA were left for further study. IgE isotype was discarded completely due to a very small number of reads). we filtered out patient 8 in the NP group from subsequent analysis, as we recognized after the run, that he had developed EBV+ post-transplant lymphoproliferative disease (PTLD) at 2.2 yrs post Ktx
Replication	We replicate our results considering Recon and downsampling. We also replicate our gDNA data using the cDNA samples.
Randomization	The subjects in this study come from a clinical trial where subjects were randomized (1:1) to a traditional low dose steroid-based immunosuppression regimen (steroids, standard daclizumab induction until the second month post-transplant, and maintenance immunosuppression with tacrolimus (Prograf, Astellas Pharma) and MMF (CellCept, Hoffman-La Roche) or a steroid-free immunosuppression regimen (prolonged daclizumab induction until the sixth month post-transplant, tacrolimus and MMF).
Blinding	Three clinical phenotype groups, defined by blinded central pathology reads of serial allograft biopsies scored by Banff criteria and the chronic allograft damage index (CADI) score were considered in this study: Non-progressors (NP; n=10) had low non-incremental CADI score without acute rejection, progressors with no rejection (PNR; n=10) had incremental CADI score over 2 yrs without rejection, and progressors with rejection (PR; n=7) had incremental high CADI scores over 2 yrs with rejection episodes

Reporting for specific materials, systems and methods

We require information from authors about some types of materials, experimental systems and methods used in many studies. Here, indicate whether each material, system or method listed is relevant to your study. If you are not sure if a list item applies to your research, read the appropriate section before selecting a response.

Materials & experimental systems

n/a	Involved in the study	
☒	☐	Antibodies
☒	☐	Eukaryotic cell lines
☒	☐	Palaeontology
☒	☐	Animals and other organisms
☐	☒	Human research participants
☐	☒	Clinical data

Methods

n/a	Involved in the study	
☒	☐	ChIP-seq
☒	☐	Flow cytometry
☒	☐	MRI-based neuroimaging

Human research participants

Policy information about studies involving human research participants

Population characteristics	The subjects were classified into three groups as described above (NP, PNR and PR). Mean value of donor age was 30 and recipient 12. Gender donor was 14 male and 13 female and gender recipient 16 male, 11 female. A total of 13 grafts came from cadaver and 14 from living related. The race of the recipients were distributed as 12 Caucasian, 6 African American, 1 Asian and 8 Other. They were allocated to two types of immunosuppression: 13 steroid based and 14 steroid free. they had a mean value of 4 HLA mismatches and the cause of end stage renal disease was distributed as: 4 patients has a non-immune structural mediated, 13 were other/unknown and 10 had a reflux disease. None of the characteristics were significantly different by clinical outcome and therefore they were not potential confounding factors, nevertheless the statistical analysis are adjusted by all tof them.
Recruitment	Pediatric subjects, age 0 to 21 years, who received a primary kidney transplant from a deceased or living donor, were enrolled following IRB approval, informed consent and, where appropriate, patient assent. This multi-center study used a randomized, open-label, parallel group design. Treatment with steroids within the 6 months prior to transplantation, en bloc kidney transplantation, high panel reactive antibody (PRA) levels (>20%), pregnancy, transplantation of a solid organ or bone marrow or hematopoietic stem cell transplant in addition to a kidney, HIV positivity/AIDS, hypersensitivity to tacrolimus, mycophenolate mofetil (MMF), prednisone, Cremophor, HCO-60, or murine products, and the inability to measure height accurately were

	exclusion criteria. Donor kidney exclusion criteria included donor age >55 years, kidney donation after cardiac death, cold ischemia time >20 h for simple cold storage, and expected maximum cold ischemia time >30 h for perfusion preservation.
Ethics oversight	IRB approval

Note that full information on the approval of the study protocol must also be provided in the manuscript.

Clinical data

Policy information about clinical studies

All manuscripts should comply with the ICMJE guidelines for publication of clinical research and a completed CONSORT checklist must be included with all submissions.

Clinical trial registration	NCT00141037
Study protocol	SDY132 ImmPort
Data collection	Patients will participate in this study for 3 years. Participants will be randomized (1:1) to one of two groups. The study includes 23 study visits over 3 years. A physical exam, medication history, adverse events reporting, blood pressure readings, growth assessment, and blood collection will occur at most visits. At the time of transplantation, participants will have a kidney biopsy. Participants will also undergo cataract screening within 4 months of transplantation.
Outcomes	1. The Difference in Linear Growth by Treatment Assignment at 1 Year Post Kidney Transplantation [Time Frame: One year post kidney transplantation procedure]<br>Standardized Z-scores were computed following a formula using an age- and gender-specific calculation provided by the NHANES III 2000 Growth Data set. The Z-score system expresses anthropometric values of height as several standard deviations (SDs) below (e.g., a negative value) or above (a positive value) the reference mean or median value. In this study the measure was used to test whether there is a difference in the change in height between the treatment groups: Steroid-Based versus Steroid-Free<br><br>2. Comparison by Treatment Assignment in the Number of Biopsy-Proven Acute Rejections Within 12 Months Post Kidney Transplantation [Time Frame: Up to one year post kidney transplantation procedure]<br>Biopsy-proven acute renal (kidney) rejection [1, 2].
