# Supplementary material for: Characterizing pre-transplant and post-transplant kidney rejection risk by B cell immune repertoire sequencing
Source: Nat Commun. 2019 Apr 23;10:1906. doi: 10.1038/s41467-019-09930-3 (PMC6479061; doi:10.1038/s41467-019-09930-3)
Supplement: Supplementary file 4 — Description of Additional Supplementary Files [file 41467_2019_9930_MOESM4_ESM.pdf]

Description of Additional Supplementary Files

Supplementary Data 1

The primers used with MiSeq (M154 and M155) and 454 Titanium (T7).
